# Photoluminescence from NV^−^ Centres in 5 nm Detonation Nanodiamonds: Identification and High Sensitivity to Magnetic Field

**DOI:** 10.1186/s11671-019-3111-y

**Published:** 2019-08-16

**Authors:** Vladimir Yu. Osipov, François Treussart, Soroush Abbasi Zargaleh, Kazuyuki Takai, Fedor M. Shakhov, Benjamin T. Hogan, Anna Baldycheva

**Affiliations:** 1 Ioffe Institute, Polytechnicheskaya 26, St. Petersburg, 194021 Russia; 2 Laboratoire Aimé Cotton, CNRS, Université Paris-Sud, ENS Paris-Saclay, Université Paris-Saclay, 91405 Orsay, France; 3 Department of Chemical Science and Technology, Hosei University, 3-7-2, Kajino, Koganei, Tokyo, 184-8584 Japan; 4 Department of Engineering, University of Exeter, Exeter, EX4 4QF UK

**Keywords:** Nanodiamonds, Nitrogen-vacancy centres, Luminescence quenching, Electron paramagnetic resonance, Magnetic field, Ground state spin levels anti-crossing

## Abstract

The content of nitrogen-vacancy (NV^−^) colour centres in the nanodiamonds (DNDs) produced during the detonation of nitrogen-containing explosives was found to be 1.1 ± 0.3 ppm. This value is impressive for nanodiamonds of size < 10 nm with intentionally created NV^−^ centres. The concentration was estimated from the electron paramagnetic resonance as determined from the integrated intensity of the *g* = 4.27 line. This line is related with “forbidden” ∆*m*_s_ = 2 transitions between the Zeeman levels of a NV^−^ centre’s ground triplet state. Confocal fluorescence microscopy enables detection of the red photoluminescence (PL) of the NV^−^ colour centres in nanoscale DND aggregates formed from the 5-nm nanoparticles. Subwavelength emitters consisting of NV^−^ with sizes a few times smaller than the diffraction-limited spot are clearly distinguished. We have further observed an abrupt drop in the PL intensity when mixing and anti-crossing of the ground and excited states spin levels in NV^−^ occurs under an applied external magnetic field. This effect is a unique quantum feature of NV^−^ centres, which cannot be observed for other visible domain light-emitting colour centres in a diamond lattice.

## Background

Fluorescent nanodiamonds (NDs) containing nitrogen-vacancy (NV^−^) centres are novel nanomaterials that open the way to innovative applications. Specific applications currently include magnetic sensing [1], bioimaging [2], and telecommunications and information processing, including the use of nanoresonator-coupled photon sources [3, 4]. The wide range of fields of application arises from the unprecedented unique quantum properties of the NV^−^ colour centres occurring at room temperature. The colour centres possess triplet spin properties that can be detected by optically detectable magnetic resonance (ODMR) [5]. The fluorescence properties of the nanodiamonds combined with their small sizes (< 40 nm) enable their use in biomedical applications including the intra-cell contrast imaging of submicron organelles and nanometre-scale thermometry in embryos [6–8]. Encapsulation of nanodiamond crystallites in biocompatible translucent shells is especially promising for their application in biological environments [9]. They can also be used as point-like probes for measurement of the Johnson noise in metals [10]. Nanodiamonds can be produced during the detonation of explosives, giving so-called detonation nanodiamonds (DNDs). However, DNDs typically contain a high concentration of imperfections. This limits the application, for example, of DNDs (size < 6 nm) in magnetic sensing applications and other local probings at the nanoscale [11].

A DND is synthesized from the pyrolysis products of the explosives’ energy-releasing components during detonation. The explosive, and detonation, is contained within a water reservoir. The synthesis takes place during a time period of, for example, 13–20 μs during the detonation of a block of explosives up to 10 cm in size. This time is that taken for the detonation wave to propagate through the entirety of the explosive block. However, each individual explosive microgranule decomposes and gasifies in a much shorter time (< 0.1 μs). Carbon and nitrogen are the predominant components of explosive substances. During the detonation, diamond crystallites up to 5 nm in size are self-assembled from the decomposition products of the explosive components, i.e. predominantly carbon. Nitrogen also incorporates into the diamond lattice during the synthesis. The presence of CH_3_* radicals in the gas-phase decomposition products causes a rapid diamond-lattice assembly in a complex C–N–O–H gas environment. The gas environment changes on a microsecond scale, with fast pressure unloading and temperature drops giving environmental conditions corresponding to the required region for diamond synthesis and stability on the Р–Т phase diagram of carbon [12].

The extremely rapid assembly of diamond crystallites in condensation centres (and also non-diamond forms of amorphous carbon) causes the appearance of a large number of imperfections, vacancies and multi-vacancies of a priori different morphologies within the growing lattice. Vacancies are naturally present as the result of stacking faults and inaccuracies that occur during the rapid assembly of a diamond lattice. During the reduction of the temperature, from the maximum during the detonation down to 800–900 °C, vacancies can still move within the lattice. The vacancies can therefore unite into clusters or disappear at the surface of the crystallites. The mobility of the vacancies at this stage of the synthesis also allows their capture by substitutional nitrogen atoms. The same processes also apply to nitrogen impurities, with the only difference that isolated nitrogen substitutions are practically immobile in the lattice at temperatures below 1600 °C and hence aggregate only at high temperatures. Nitrogen impurities can be present within the lattice of DNDs in different forms. It can be present in an aggregated form (A-centres, dimers) in an amount up to ~ 2.5 at.% or in the form of isolated carbon-substituted atoms (C-centres). As is well known, A-centres are non-paramagnetic and have spin zero, whereas C-centres with spin-half are paramagnetic in the neutral form (P1-centres). Therefore, P1-centres can be easily detected by the Electron Paramagnetic Resonance (EPR) method. A vacancy in the lattice, being negatively charged (V^−^), is also paramagnetic but has spin 3/2. Spin-half spins were found in DNDs in the prevalent amount [13]. The concentration of spin-half radicals detected by the EPR method is practically the same (1100–1400 ppm)^1^ in all standard DNDs of industrial production. This is irrespective of the specific commercial producers and details of their technology which can differ in the finer details. The spin-half radical concentration is approximately the sum of the total number of substitutional nitrogen atoms and other defects having dangling bond spins within the DND particle compared to the particle weight (or the corresponding number of carbon atoms in it). The majority of the nitrogen (up to ~ 2.5 at.%) in DNDs is distributed more or less homogeneously in the covalent lattice of the diamond cores but enriched at defect regions such as stacking faults [14, 15]. It was also concluded by Turner et al. that the embedded nitrogen is predominantly present in the diamond lattice in the form of N–N dimers (A-centres) or isolated neutral, positively or negatively charged nitrogen *sp*^3^-coordinated impurities (C-centres) [14, 15]. Thus, most of the nitrogen in a DND is in the non-paramagnetic form of nitrogen pairs (N–N) located in adjacent nodes of the diamond crystal lattice.

In addition to prevalent nitrogen impurities, DND particles also contain isolated vacancies, multivacancies, nitrogen-vacancy-nitrogen (NVN) and nitrogen-vacancy (NV)-type defect centres. These additional defect centres occur due to the spontaneous appearance of the constituent agents in neighbouring lattice sites during the lattice assembly [11]. At the same time, the presence of negatively charged NV^−^ sites is mainly due to their charging and accepting the negative charge from excess neutral nitrogen atoms playing the role of electron donors in the system. According to our preliminary EPR data, the concentration of NV^−^ centres which can be determined from the integrated intensity of the EPR line with a *g*-factor of 4.26–4.27 in a half magnetic field is about 2.5–3 orders of magnitude less than the concentration of the isolated substitutional nitrogen, giving a value of 400–700 ppm.

Multivacancies and NV^−^ centres were successfully identified in DNDs by the EPR method in our previous recent work [13, 16, 17]. Efforts have also been made to understand the relationship between the brightness of NV^−^ centres in DNDs and the chemical composition of their surface, their environment and the morphology of aggregates [18, 19]. Similar work on controlling the emission properties of NV^−^ was also done for synthetic fluorescent diamonds with artificially created NV centres [20]. Nevertheless, necessary studies combining different analysis methods, including X-ray powder diffraction, EPR spectroscopy, confocal fluorescence spectroscopy and microscopy, elemental analysis, analysis of emitters of diffraction-limited sizes, and magnetic field influence on the photoluminescence signal have not been fully performed on the topic of NV^−^ centres in DNDs except in our preliminary work [21]. Currently, the dependence of emission properties of NV^−^ in DNDs upon the interior impurity content and particle morphology is not understood. In this work, we report that DND particles demonstrate specific photoluminescence (PL) of the NV^−^ centres, precisely count the content of NV^−^ in them via a sophisticated approach, and analyse their PL intensity at the spectral maximum as a function of diamond crystallite size and nitrogen content. Furthermore, we demonstrate the DNDs’ sensitivity to an external magnetic field which relates to their ODMR properties.

## Experimental

### Fabrication Methods and Controlling the DND Morphology

DND powders were obtained by a largely standard process which involves the blasting of around ~ 1 kg of an explosive mixture of trinitrotoluene-hexogen surrounded by a water shell of appropriate thickness, in a closed stainless steel vessel of several cubic metres. This is a so-called “wet” synthesis. Synthesis and primary purification of the product was carried out by a commercial manufacturer—the Special Design and Technology Bureau “SCTB Technolog”, St. Petersburg, Russia. The technological process used at this enterprise, makes it possible to obtain DND powders exclusively consisting of nano-crystallites with the size of the coherent scattering region (CSR) varying in the range from 4.5 nm to around 5.7 nm. Some purified DND powders prepared by means of standard technology were supplied by PlasmaChem GmbH (Berlin, Germany). The morphology of as-delivered DND samples was established by X-ray diffraction (XRD). XRD patterns were recorded in Hosei University Micro- and Nanotechnology Research Center (Tokyo, Japan) by means of a Rigaku SmartLab I X-ray diffractometer with D/teX Ultra detector, CuKα radiation source (*λ* = 1.54178 Å) and nickel filter in the angle range 2Θ = 5–100^o^ (step 0.01^o^). The width of the hardware function was taken into account in the course of analysis. X-ray CSR sizes were determined on the basis of sophisticated analyses of the widths of 111, 220 and 311 reflexes as a function of 2Θ angle in accordance with the method described in Ref. [22].^2^ The key feature of this method is that the lattice constant parameters (*a*_o_ = 3.5640 Å) can be obtained for all DND samples with systematic errors (variations due to the choice of different models) no greater than ± 0.0003 Å. X-ray powder diffraction measurements demonstrated a CSR size of one representative DND sample chosen for subsequent studies of 5.2 ± 0.2 nm. This DND sample was used in all the main studies described below. Additionally, a few DND samples synthesized by the same manufacturer under special conditions (with and without inorganic additives to the detonating zone) and having different X-ray CSR sizes were used for plotting the dependence of the photoluminescence intensity versus the CSR size or/and the content of interior nitrogen. The CSR size of DND powder synthesized by ordinary technology was 4.6 ± 0.2 nm.

The DND powders were further purified in boiling acid mixtures to remove any residual impurities of 3d ferromagnetic metals, thus allowing for more accurate EPR and XPS studies. Additional surface cleaning by annealing the DND powders in the air at 430 °C for 10–12 h was required for Raman scattering and subsequent photoluminescence studies [23]. No further effort was made to de-aggregate the DND powder containing individual discrete nanodiamond particles and aggregates with sizes below 25 nm after having been acid-purified and treated in air. The mean size of DND aggregates in water suspension was 25–30 nm.

Samples for confocal fluorescence microscopy studies were fabricated from aqueous suspensions of the DNDs. Suspensions were obtained by dispersing the acid-purified and air-annealed powder in water at a concentration ≈ 1 mg/ml, with subsequent dilution in water by a factor of 100. The DND suspensions were then further spin-coated onto a 170-μm-thick cover-glass substrate, which was pre-cleaned in a mixture of ethanol and acetone in an ultrasonic bath. Finally, DND aggregates deposited on cover-glass substrates were cleaned with a UV/O-Cleaner (low-pressure mercury lamp: optical power > 25 mW/cm^2^ at *λ* = 254 nm) for around 30 min. Under UV light exposure, the efficient oxidation and gas-phase etching of the light-absorbing *sp*^2^*-*carbon around the DNDs with oxygen and ozone occurs. The exterior nanoscale clusters of *sp*^2^-carbon are extremely undesirable for studying the PL from the intrinsic colour centres in the diamond lattice of DNDs. Exposure of the substrate to UV radiation also leads to the removal of any unwanted organic contaminants, which can otherwise give rise to a luminescent background in a wide spectral range.

### Characterization Methods

#### Magnetic Resonance Spectroscopy

**Fig. 1:**
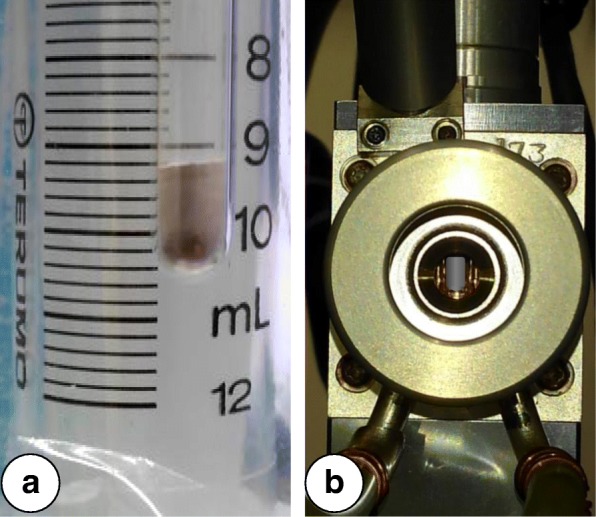
Photographs of DND powder in **a** the JEOL EPR tube and **b** in the centre of X-band microwave cavity. For comparative purposes, the image of the powder in the EPR tube is specially taken against the main part of a plastic syringe with a volume of 10 ml

The EPR spectra of the samples were recorded at room temperature, within the microwave X-band at a requency of ~ 9.4393 GHz or ~ 9.0785 GHz, using an EPR spectrometer (JEOL JES-FA 300 (Japan)). The particular fixed frequency used depended upon the presence or absence of a quartz adiabatic cryostat in the microwave cavity during the whole research cycle. Dozens of milligrammes of DND powder were introduced into a long X-band JEOL EPR tube (JEOL Parts Catalogue, item 4220 00281, No.193) with an outer diameter of 5 mm and 100 mm in length quartz bottom side. The open top of the tube was sealed against moisture. Photographs of the DND powder in the JEOL EPR tube and in the centre of the microwave cavity are shown in Fig. 1 a and b. The powder, of grey colour, is clearly seen in both panels. Such an amount of DND powder is suitable both for effective tuning of the EPR spectrometer with high Q-factor, and obtaining a good signal-to-noise ratio during recording the spectra of weak signals in the half magnetic field region.

The EPR spectra of the DND, with *g*-factors of the signals in the range 4.00–4.30, were recorded with microwave power, *P*_MW_ = 6 mW; magnetic field modulation amplitude, *A*_m_ = 0.5 mT; frequency, *ν* = 100 kHz; gain, *G* = 1200; and signal accumulation, *N* = 20. The time constant was 0.030 s. The recording time for one magnetic field sweep was 120 s.

The minimal and maximal magnetic field values for the sweep were specially selected with the JEOL-JES program software and precisely defined with a JEOL NMR Field Meter ES-FC5 (Echo Electronics). The concentration of NV^−^ centres was estimated by double integration of the corresponding EPR signal with *g* = 4.27, followed by assignment to the corresponding sample weight and comparison to the EPR signal from a reference sample. We specially chose a synthetic fluorescent Ib HPHT diamond powder^3^ (mean size ~ 100 nm) with a well-defined integrated intensity of the *g* = 4.27 EPR line and known concentration of *S* = 1 NV^−^ centres of 5.3 ± 0.4 ppm as an independent, reliable reference to enable correct determination of the obtained concentration of NV^−^ in the DND. This is similar to the approach proposed previously by Shames et al. [24]. The *g* = 4.27 EPR line was recorded for this reference sample with the following parameters: *P*_MW_ = 3 μW, *A*_m_ = 0.2 mT, *G* = 500, and *N* = 12 in a linear mode where the peak-to-peak intensity of the EPR line is proportional to the square root of the microwave power in the sample space. For submicron and micron-sized Ib HPHT crystals, this regime is usually well-enforced at low microwave powers (below 7–10 μW). The spectrum was specially recorded in the wide range of magnetic field from 119 to 321 mT. This range was used in order to register additional EPR signals from Δ*m*_s_ = 1 transitions, which are allowed with low-field, and represent unique signatures of the NV^−^ centres.

#### X-ray Photoelectron Spectroscopy

X-ray photoelectron spectroscopy (XPS) of DNDs was performed using a Perkin-Elmer PHI 5600 Multi-Technique system, equipped with a source of monochromatic Al Kα radiation. The spectral characteristics of photoelectrons were obtained after analysing the photoelectron flux emitted from a sample area of ~ 1 mm^2^ [25]. The detected photoelectrons are emitted from the material’s surface—a few atomic-layer thick regions (< 2 nm). Particular attention was paid to analyses of the areas of photoemission peaks of carbon C1s, nitrogen N1s and oxygen O1s. Decomposition of the photoemission peaks into individual components was performed by an appropriate set of singlet contours. Spectra of the DND sample were taken after etching of the surface with argon ions. Ion etching was carried out for 3 min at an accelerating voltage of 3 kV and with an ion beam current of around 4 μA. The Ar ion etching was used to remove adsorbed ambient gases and other chemisorbed atomic complexes from the sample surface. XPS was also used for evaluating the interior nitrogen content in a series of different DND samples with different X-ray CSR sizes.

#### Fluorescence Spectroscopy

For PL studies, samples were prepared by pressing the powder flush into a shallow hole with a diameter of 2 mm made in a 4-mm copper plate, followed by levelling of the surface with a glass plate. PL spectra were recorded in the range 540–1000 nm by means of a custom micro-Raman setup with a 532-nm laser excitation at a power of ~ 0.5 mW and diffraction grating with 600 grooves per millimetre. The exposure time was 180 s. The excitation radiation was focused on the flat sample surface through a Nikon Plan Apo 100×, NA = 1.40 microscope objective into a spot of 2 μm in diameter. The spectral resolution was better than 0.05 nm.

#### Confocal Fluorescence Microscopy

To register the PL spectra and map the PL signal from DND particles spin-coated on cover-glass, we used a further PL setup. It consists of a custom inverted confocal scanning microscope combined with an imaging spectrograph. The confocal microscope includes a piezoelectric scanning stage (NanoPDQ75, Mad City Labs Inc., USA), a microscope objective (Plan Apo 100×, NA = 1.40, Nikon, Japan) and an optically pumped semiconductor laser (Verdi, Coherent Inc., USA) emitting at a wavelength of 532 nm that is injected (circular polarization, 0.5 mW power) into the objective through a dichroic beam splitter (z532rdc, Semrock, USA). The detection path consists of a 50-μm-diameter pinhole for confocal rejection, a bandpass filter (FF01-697/75, Semrock) and a single-photon-counting module detector (SPCM-AQR-14, Perkin-Elmer, Canada). The imaging spectrograph is composed of a concave grating imaging the spectrum on a back-illuminated CCD array detector (DU440-BU2, Andor Technologies, UK), leading to a spectral resolution of about 1 nm.

Mapping of the luminescence signal was carried out over a 20 × 20 μm^2^ square. The elementary stage displacement in lateral directions was 100 nm at accuracy ~ 1 nm. To obtain PL spectra in the range of 500–900 nm and time dependence of the PL intensity at the maximum of the appropriate spectrum, two of several DND aggregates found on the substrate with lateral sizes no more than 0.6 microns and having intense luminescence were selected. The exposure time was 60 s for each PL spectrum and 3 ms per pixel in each registered 2D PL map. The diffraction-limited spot size (point spread function, PSF) covers about 2–3 pixel (250 nm). The magnetic field was applied using a permanent magnet brought to within around 1 mm of the cover-glass substrate. The magnetic field strength at the sample location was around ~ 90–100 mT. The time dependence of the PL signal was recorded with repeated rapid approach and removal of the magnet to and from the surface with the sample.

## Results and Discussion

### Counting NV^−^ Centres in DND by Half-Field EPR Method

**Fig. 2:**
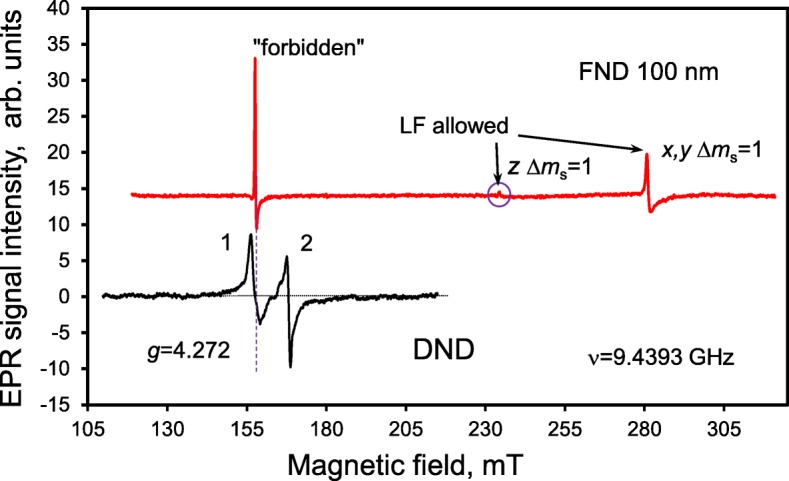
EPR spectra of acid-purified DNDs in the region of half magnetic field (black curve) and of reference electron-irradiated fluorescent Ib HPHT diamond nanoparticles with mean size ~100 nm (red curve) in the range up to 320 mT. Both lines of the DND EPR spectrum are marked by numbers 1 and 2. The low-field line 1 with *g* = 4.272 corresponds to NV^−^ centres. Lines related with low-field (LF) allowed *z* Δ*m*_s_ = 1 and *x*,*y* Δ*m*_s_ = 1 transitions in FND 100 nm are marked by arrows for the upper spectrum. The weakest, barely distinguishable line related with allowed *z* Δ*m*_s_ = 1 transition is additionally marked by a circle. The microwave frequency was 9.4393 GHz

**Fig. 3:**
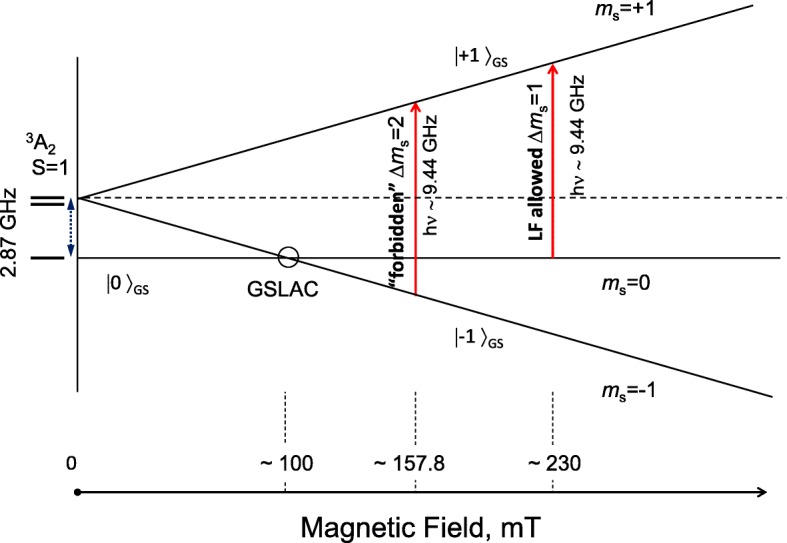
Energy diagram of ground singlet-triplet levels ^3^A_2_ of NV^−^ in magnetic field up to 300 mT. “Forbidden” Δ*m*_s_ = 2 and LF allowed Δ*m*_s_ = 1 transitions caused by absorption of microwave radiation (*ν* ≈ 9.44 GHz) are marked by vertical red arrows. The position of the ground state spin level anti-crossing (|0 〉_GS_ and |−1〉_GS_) is marked by a circle

**Fig. 4:**
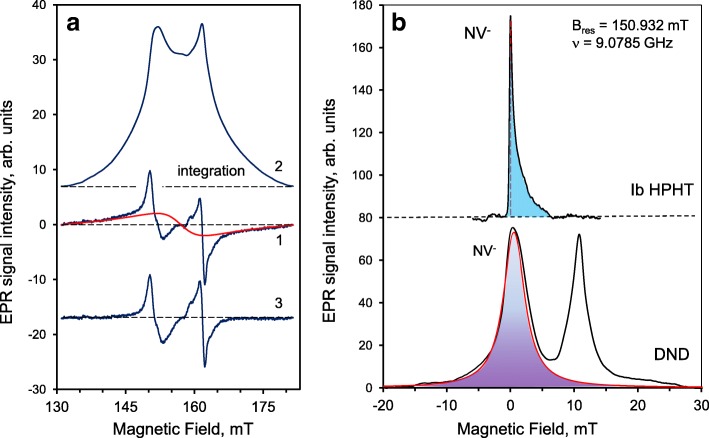
**a** Background subtraction in EPR signal of DND and **b** estimations of the double-integrated intensities of the *g* = 4.27 line for a DND sample and reference fluorescent Ib HPHT synthetic diamond. Panel **a**: as-registered first derivative EPR signal of the DND in the ± 15 mT half magnetic field range (curve 1, blue); the same, but integrated EPR signal in the same ± 15 mT range of magnetic field (curve 2); the same first derivative EPR signal of DND, but after subtraction of the broad parasitic EPR signal from remaining non-removable iron-containing paramagnetic complexes shown by red contour of Lorentzian shape in the upper curve (curve 3, blue). Panel **b**: Estimation of the double integrated intensity of the *g* = 4.27 line for a fluorescent Ib HPHT diamond having NV^−^ (upper curve, shaded area) and the DND sample (bottom curve, shadow area). The bottom spectrum in panel **b** consists of two contours of Lorentzian shape, one of which centred at lower magnetic field (≈ 150.932 mT), is assigned to the NV^−^ centres of DND (the area below this contour in red is highlighted). Microwave frequency: ν = 9.0785 GHz

Since the radiation emitted by colour centres can be absorbed by *sp*^2^-containing species present inside the DND aggregates and may even be blocked by particular morphological features in the space between the particles, it is very important to control and monitor NV^−^ centres in diamonds by non-optical methods. The NV^−^ centres can be detected by EPR spectroscopy even when embedding their carriers into the non-transparent, dense medium which blocks both the exciting and luminescent optical radiation or in the case of the absence of NV^−^ secondary radiation related with specific quenching mechanisms. Their total concentration can subsequently be easily evaluated by analysis of the EPR spectrum [16, 26, 27]. At the same time, the determined concentration of NV^−^ centres provides valuable information on the potential (after extensive purification) brightness of the NV^−^ PL. The EPR spectrum of the DND powder purified from 3d transition metal magnetic impurities (mostly Fe, Ni) in the region of the so-called half magnetic field is shown in Fig. 2 (black curve). The spectrum consists of two close lines (1 and 2) with *g*-factors *g*_1_ = 4.27 and *g*_2_ = 4.00, and widths *ΔH*_pp1_ ≈ 2.0 mT and *ΔH*_pp2_ ≈ 1.4 mT. The first, low-field line, corresponds to the forbidden transitions Δ*m*_*s*_ = 2 between the Zeeman energy levels of the triplet state of the NV^−^ centre in a magnetic field. The second, high-field line, corresponds to the same forbidden transitions Δ*m*_*s*_ = 2, which occur when microwave radiation is absorbed by other centres, i.e. multivacancies having *S* = 1. In Ref. [16], these lines were first assigned to different triplet centres coexisting in the DNDs, and in particular, the *g*_1_ = 4.27 line was assigned to a NV^−^ centre. The position of the *g*_1_ = 4.27 line corresponded to that of the forbidden transitions Δ*m*_*s*_ = 2 as shown in the energy diagram of the ground state of the singlet-triplet NV^−^ centre in magnetic fields up to 300 mT (Fig. 3). A large zero-field splitting parameter of the spin-Hamiltonian (*D* = 850 × 10^−4^ cm^−1^), caused by the strong exchange interaction of the constituent spins in the triplet NV^−^ centre, causes the high sensitivity of the position of the *g*_1_ = 4.27 line (shifting by 0.5% on the *g*-scale) to the specific microwave frequency in the range from 9.0 to 9.9 GHz (X-band) [26]. The integral intensity of the line *g*_1_ = 4.27 can be used to precisely estimate the concentration of NV^−^ centres even when the luminescence of the nanodiamonds is not detectable due to absorption or quenching due to neighbouring optically active species, or even masking by strong parasitic background luminescence from the third co-existing species. A high-energy electron-irradiated, synthetic, submicron diamond (mean size 100 nm) with a known concentration of NV^−^ centres was used as a reference sample (sample Ib HPHT FND). The EPR spectrum of sample Ib HPHT FND taken at low microwave power *P*_MW_ = 3 μW is shown in Fig. 2 (red curve) for comparison. In the range below 320 mT, it consists of three well-defined EPR signals corresponding to a “forbidden” Δ*m*_s_ = 2 (at 157.85 mT), and two allowed *z*, Δ*m*_s_ = 1 and *x*,*y*, Δ*m*_s_ = 1 (at 234.39 mT and 281.27 mT) transitions between the Zeeman energy levels of the ground triplet states of NV^−^ in magnetic field. The Δ*m*_s_ = 2 and *z*, Δ*m*_s_ = 1 transitions are also shown on the scheme in Fig. 3. It is fairly surprising that in comparison with the submicron reference sample with NV^−^ centres, the DND particles demonstrate only the *g* = 4.27 EPR line related with Δ*m*_s_ = 2 “forbidden” transitions. Notably, the allowed *z*, Δ*m*_s_ = 1 and *x*,*y*, Δ*m*_s_ = 1 microwave transitions are not observed. A probable explanation is the abnormal broadening of the EPR lines related with Δ*m*_s_ = 1 transitions due to the large variation in the main parameters of the spin-Hamiltonian (*D* and *E*) for the *S* = 1 NV^−^ over the ensemble of DND particles. Thus, the complete absence of lines related with the allowed *z*, Δ*m*_s_ = 1 and *x,y,* Δ*m*_s_ = 1 transitions is a key unconventional feature of NV^−^ in DNDs. According to the commercial supplier, the expected concentration of NV^−^ in the reference Ib HPHT FND evaluated on the basis of the absorbed irradiation dose was ~ 5.3 ppm. The integral intensity of the *g*_1_ = 4.27 line in the DNDs’ EPR spectrum was found to be ~ 4.8 times smaller than that in the Ib HPHT FND EPR spectrum, and therefore, the concentration of NV^−^ centres in the DNDs is determined to be 1.1 ± 0.3 ppm. The corresponding procedures of integration of both the EPR signals in the half magnetic field range (130–180 mT) together with background correction are specially shown in Fig. 4. Figure 4 a contains the original, as-measured EPR spectrum of the first derivative of the microwave absorption, prior to the mathematical treatment (curve 1); the same EPR spectrum after the integration (upper curve 2); and the same as-measured original EPR spectrum, but after subtracting the broad Lorentz-type line related with remaining iron-containing complexes (bottom curve 3). Figure 4 b shows the EPR spectra of both the reference sample (upper curve) and DND (bottom curve) after integration of the background-corrected, as-measured EPR spectra. The true integrated spectrum for the DND itself (Fig. 4b, bottom curve) is quite different from the second spectrum in Fig.4a, where only the uncorrected, as-measured EPR spectrum of DND was integrated. The obtained spectrum (Fig. 4b, bottom curve) demonstrates a very deep valley between the neighbouring EPR signals (*g*_1_ = 4.00 and *g*_2_ = 4.27) of Lorentz-type separated by ~ 10 mT distance. The shaded areas under the EPR spectra shown in panel (b) of Fig. 4 give the integral intensities of the line *g*_1_ = 4.27 for both compared samples. The ratio of these areas when normalized by sample weights is about ~ 4.8. Interestingly, for the DND, the shape of the line *g*_1_ = 4.27 is symmetric and broad. This line is partially superimposed with the high field *g*_2_ = 4.00 EPR line caused by multivacancies. Some remaining uncorrected background in the form of a strong baseline (mainly from non-removable iron-related complexes) is clearly seen in Fig. 4a (spectrum 2). Thus, correct decomposition of the EPR spectrum of DND for at least three overlapped components in the range 130–180 mT is a primary need for correct evaluation of the integrated intensity of the *g* = 4.27 line and concentration of NV^−^. We also found that various treatments at temperatures below 500 °C (in vacuum and in air) have no practical effect on the concentration of NV^−^ centres in the DNDs. The double-integrated intensity of signal *g*_1_ = 4.27 (shadow area below the Lorentzian contour centred at ~ 151 mT for *ν* = 9.0785 GHz) remains largely the same after treatment as before. This indicates that the NV^−^ centres are buried sufficiently deep in the lattice, at a depth from the nanoparticle surface of at least the lattice constant (~ 0.36 nm), that they therefore cannot react with external chemical agents that do not etch or penetrate the diamond phase.

The main high-intensity EPR signal of the DND lies above 320 mT at *ν* = 9.4393 GHz and has a *g*-factor *g* ≈ 2.0027. It has a Lorentzian curve profile of linewidth Δ*H*_pp_ = 0.84 mT [13]. This linewidth is greater than that of fluorescent nanodiamonds (FNDs, 100 nm) milled from microcrystalline diamond synthesized by a high-pressure and high-temperature method. The broad signal can be explained by greater exchange and dipole-dipole interactions between the *S* = 1/2 spins in the spin ensemble within an individual DND nanoparticle than those within a FND. The intensity of the main EPR signal collected from all *S* = 1/2 paramagnetic centres, both of nitrogen (P1 centres [28]) and non-nitrogen origin, indicates a spin concentration of ~ 1300 ppm, corresponding^4^ to around 15 *S* = 1/2 spins in each DND [13]. However, it can be concluded from the earlier obtained data that approximately 40–50% of the total number of all paramagnetic centres in the DNDs are due to half-populated antibonding orbitals of isolated P1 centres. Thus, the huge total of spin-half, point-like agents located in DNDs (*N*_PC_) can be represented as the sum of at least two contributions, from P1 centres and from elemental point defects having dangling bond spins ½, for example based on vacancies like H1 centres (VH^o^): $\left({N}_{\mathrm{PC}}=\left[{\mathrm{N}}_{\mathrm{s}}^{\mathrm{o}}\right]+\left[\mathrm{et}\ \mathrm{al}\ \right]\ \right)$. Here we assume that specific EPR signatures from the hyperfine structures of P1 and H1 centres are absent or greatly “smeared” through the dense arrangement of localized spins within each particle. This sum gives us a clue about the approximate content of both isolated nitrogen and monovacancies inside the DND, although isolated nitrogen and monovacancies can also be present in the diamond lattice in nonparamagnetic forms such as ${\mathrm{N}}_{\mathrm{s}}^{-},{\mathrm{N}}_{\mathrm{s}}^{+}$, V^o^. The presence of H1 centres together with neutral monovacancies in DND at some minor level (< 700 ppm) is reasonable in principle because rapid assembling of diamond lattice during the detonation takes place from hydrogen-containing products of TNT-hexogen pyrolysis such as CH_3_^*^ or CH_2_^*^ radicals. Although the mutual charge transfers between the main groups of centres present in the lattice in various charge and spin states (${\mathrm{N}}_{\mathrm{s}}^{\mathrm{o}},{\mathrm{N}}_{\mathrm{s}}^{-},{\mathrm{N}}_{\mathrm{s}}^{+};{\mathrm{V}}^{-},{\mathrm{V}}^{\mathrm{o}}$) are possible in principle, the foremost contribution to paramagnetism comes from only ${\mathrm{N}}_{\mathrm{s}}^{\mathrm{o}}$. Let us therefore estimate the maximal possible concentration of NV in a DND on the basis of assumptions about the known total amounts of substitutional nitrogen and monovacancies inside the particles. The actual charge state of NV is not essential for such an estimation. A statistical approach gives the following simple formula for the probability (*p*_NV_) of finding at least one NV in one 5-nm DND particle consisting of *N* nodes of covalent diamond lattice: ${p}_{\mathrm{NV}}=\frac{2 nv}{N}$ , where *n* and *v* are the mean numbers of isolated nitrogen atoms and monovacancies inside the DND particle, respectively. Because *N*_PC_ ≈ 1300 ppm, we can approximately assume that *n* + *v* ~ 15 for *N* = 11,500. The maximal value of *p*_NV_ is achieved when $n\approx v\approx \frac{15}{2}$. This gives *p*_NV_ ≈ 0.0098. This value corresponds to around ~ 1 ppm of NV in DND, as was obtained previously from comparison of the *g* = 4.27 EPR signals of the DND and the reference sample. Excluding the surface nodes linked with surface functional groups and interior nodes occupied by A-centres (up to 2 at.%) from the formal integration procedure, using *N* = 9950–10,000 gives a slightly greater concentration of NV, up to 1.1 ppm. The estimated experimental concentration of NV^−^ centres in the DNDs is in close agreement with the theoretical estimation made above, and about 1000 times smaller than the concentration of all *S* = 1/2 paramagnetic species in the system.

The concentration ratio for interior *S* = 1/2${\mathrm{N}}_{\mathrm{s}}^{\mathrm{o}}$ and *S* = 1 NV^−^ centres is therefore qualitatively consistent with the main idea of rapid NV centre formation, that is, from the “random inclusion” of both substitutional nitrogen atoms and vacancies to the growing diamond nano-crystallites during the overall ~ 13–20 μs duration of the detonation wave propagation. Here, we intuitively assume that each NV^−^ is formed as a result of the random embedding and occurrence of impurity nitrogen atoms and vacancies in the nearest neighbour lattice sites during the period of time prior to the subsequent rapid cooling of the products to temperatures of the order of ~ 500–650 °C at which the diffusion of vacancies in the lattice is practically stopped.

### Nitrogen Impurity Concentrations

**Fig. 5:**
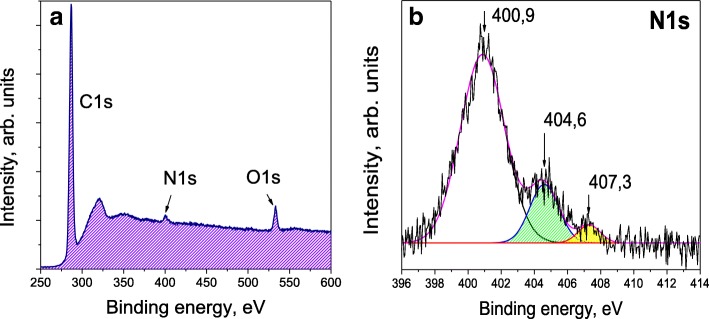
XPS spectra of acid-purified DNDs after Ar ion etching: **a** overview of the spectrum in the range 250-600 eV and **b** N1s photoemission peaks

XPS is a powerful tool for studying the DNDs’ composition and the chemical state of the main alien elements present on the surface of the DNDs (and also within 2 nm under the surface) [29]. Our main interest was focused on the XPS signal from nitrogen and the evaluation of the interior nitrogen content, since nitrogen is the predominant inner impurity. The XPS signals of carbon (C1s), nitrogen (N1s) and other elements have been recorded after etching of the surface with Ar ions. An overview of the XPS spectrum plotted in the wide range of binding energies from 250 to 600 eV is shown in Fig. 5a. Although the data indicates the presence of a large amount of oxygen-containing atomic groups at the DND’s surface (5.5 at.%), the analysis of the O1s signal is not of particular relevance to this work. The concentration of both inner and exterior nitrogen was preliminarily evaluated to be between 1.7 and 2.4 at.% [30]. The concentration of all the residual elements found (a small number of metals) did not exceed ~ 0.32 at.% in the as-received DND, and it could be reduced by 20–30 times by etching the DND powder in boiling aqua regia and hydrochloric acid [30]. The photoemission peak of nitrogen (N1s) is shown in Fig. 5b. Etching with Ar ions results in the removal of weakly bound adsorbed species from the surface, while the covalent diamond lattice remains unaffected. The characteristic high-energy peaks (~ 404.6 eV and 407.3 eV) in the N1s photoemission signal have very low intensities when compared with an untreated pristine sample. These peaks demonstrate the presence of any remaining nitrate ions (peak at ~ 407.3 eV) and nitrite groups (peak at ~ 404.6 eV) on the Ar ion-treated surface. Further complete removal of nitrate ions and nitrite groups from the surface can be achieved only by annealing the DND powder in air at temperature > 350 ^o^C. The main peak of the N1s signal at ~ 401 eV, which is not influenced by Ar ion treatment, corresponds to chemical bonds of the type N–*sp*^3^–C. This peak is a characteristic of interior elemental nitrogen covalently bound within the diamond lattice. It appears due to various forms of nitrogen present in the diamond matrix, including NN dimers, next nearest neighbouring N^+^...N^−^ pairs, and more complex nitrogen clusters. Similar data were obtained for the photoemission peak of carbon C1s (sharp, intense signal seen in Fig. 5a, see also Ref. [30] for details). The C1s XPS signal consists of two main peaks: one centred at 284.9 eV corresponding to C–C bonds in the diamond matrix and another peak centred at 287.3 eV corresponding to C–N bonds. Only diamond carbon and *sp*^3^/*sp*^2^ carbon bound to nitrogen, even in the form of atomic-scale disordered nitride phase species (where neighbouring carbon and nitrogen atoms can have up to three C–N bonds), are represented in the C1s signal of the Ar ion-treated surface. Our careful analysis of the integrated intensity of only the N1s 400.9 eV peak and C1s signals (together with O1s and residual elements’ signals) suggests that nitrogen is contained within the diamond lattice of selected DND sample in the amount of 1.65 ± 0.05 at.% and mainly in the form of complex clusters which are not paramagnetic. This value was obtained after special correction by a reducing factor taking into account that the actual size of DND particles is larger than the whole depth of crystal lattice from which the excited photoelectrons are readily emitted. It seems that only a small part of nitrogen is present in the diamond lattice in the form of isolated paramagnetic nitrogen atoms with spin *S* = 1/2 (no more than 8–9% of all nitrogen). The isolated paramagnetic nitrogen atoms are only accessible for observation by EPR as was shown in the previous section.

XPS spectroscopy was also applied to estimate the nitrogen content in other DND samples provided by our suppliers. We found that the nitrogen content varies in these samples from ~ 1.6 at.% (minimal value) to ~ 2.1 at.% (maximal value). We simultaneously noticed that higher concentrations of nitrogen correspond to the samples with a smaller X-ray CSR size. Such values of nitrogen content were also roughly confirmed by elemental analysis with a Micro Corder JMC10 device in the course of sample combustion in oxygen flow (30 ml/min) at 1000 ^o^C. As a reference source of nitrogen, carbon and hydrogen for this method, we used the antipyrine C_11_H_12_N_2_O, having nitrogen in its structure in the form of N–N groups.

### DND Fluorescence Intensity and Its Dependence upon the Nitrogen Content

**Fig. 6:**
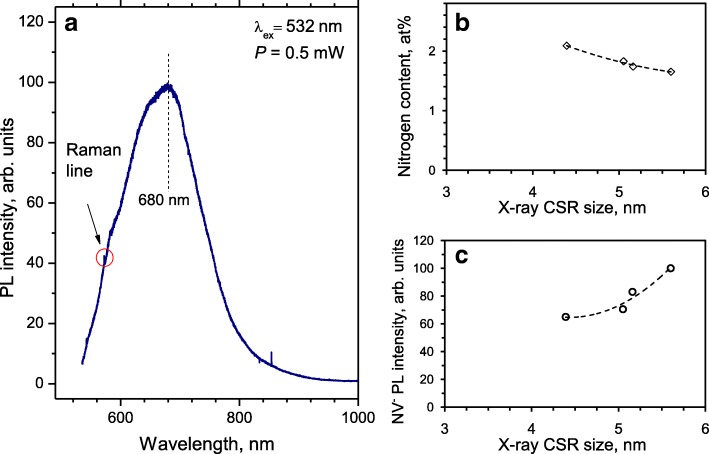
**a** Photoluminescence spectrum of DND powder pressed flush in a shallow hole with a diameter of 2 mm made in a copper plate and **b** the interior nitrogen content measured by XPS and **c** the intensity of NV^−^ PL at 680 nm as a function of X-ray CSR size for a series of selected DNDs synthesized in the presence of some intentionally added inorganic additives in the detonation zone (charge and water shell) as provided by the manufacturer. Excitation laser wavelength *λ* = 532 nm, power ~ 0.5 mW. The diameter of the focused laser spot on the sample surface was 2 μm. Conditions of recording were temperature *T* = 293 K, and an air environment

The PL spectrum of one selected powder DND sample is shown in Fig. 6a. It has the maximum intensity at 680 nm. Following [31], a characteristic spectrum with peak position above 650 nm indicates the presence of both NV^−^ and NV^0^ defects, although the contribution from some light-emitting defective sites of remaining *sp*^2^-coordinated carbon species around DND particles cannot be excluded completely. However, the characteristic zero-phonon lines (ZPL) at 638 nm (NV^−^) and 575 nm (NV^0^), respectively, are not detected. This probably occurs due to the changing positions of the NV^0^ and NV^−^ excited/ground states inside the bandgap for centres lying in the vicinity of the particle edge and the subsequent broadening of the 638-nm and 575-nm ZPL spectral components for the ensembles of such NV^0^ and NV^−^ centres with slightly different electronic parameters. Let us mark that the absence of featured NV^−^ ZPL peak at 638 nm was confirmed in many studies related with photoluminescence of DND aggregates lying free on the substrate or embedded inside polymers [31]. Sometimes it (suppressed or very poorly recognized ZPL feature at 638 nm) even happens for larger isolated fluorescent Ib HPHT nanodiamonds of a size about 40 nm [4].

We also studied the PL intensity for a series of DND samples with different CSR sizes. The CSR size characterizes the average size of perfect diamond domains or the mean size of diamond nano-crystallites in the powder even if they are randomly arranged in large-scale polycrystalline aggregates with size exceeding 30–50 nm. The CSR size varied from 4.3 to 5.6 nm in the series of DND samples selected for our studies (similar to the results explained in Ref. [32] although we used another more traditional approach for analysing X-ray diffractograms). Figure 6b shows the dependence of interior nitrogen content evaluated by means of the XPS method versus the CSR size of the DNDs. The larger the CSR size, the smaller the nitrogen content. This seems reasonable as powders with larger DND particles were synthesized as a result of the addition of some inorganic substances having the elements playing the role of nitrogen-getter inside the detonation zone (charge or charge enclosure). Such elementary additives probably promote the reduction of the overall nitrogen content in the growing diamond lattice during the explosion process or change the conditions of the diamond lattice assembly to slightly prolong the synthesis (on the order of microseconds). In addition to the overall nitrogen content, we also recorded the reduced amount of nitrogen-related paramagnetic centres in these DNDs, as confirmed by EPR spectroscopy. PL spectra of all DNDs having different CSR sizes are practically the same in shape in the range 550–900 nm, but this is not the case for the absolute intensities of the PL at the maxima of the PL spectra at 650–680 nm. The intensity at the maximum of PL spectrum is plotted in Fig. 6c as a function of the X-ray CSR size of the DNDs. Comparing both panels (Fig. 6b, c) it is clearly seen that the smaller the nitrogen content in DND, the higher the NV^−^ PL intensity. Again, this seems reasonable as nitrogen-related centres and especially A-centres can act as effective quenchers of PL if the NV^−^ light-emitting centres in some DND particles (one per a hundred of 5 nm DND particles at least) are surrounded by a “gas” of A-centres and other lattice imperfections, similar to the approach proposed in Ref. [33]. This trend gives us a hint at possible ways to enhance the intensity of luminescence from ensembles of DND particles by manipulating their size and nitrogen concentration. Among them, there is the technological enlargement of the mean size of DND particles in the course of their treatment at high pressure and high temperature at appropriate conditions, promoting their recrystallization and further crystal growth [34]. The probable reduction of NV^−^ content through treatment leading to the reduction of A-centres and other interior defects in the diamond lattice may be compensated in principle by the opposite trend, promoting the brightness of NV^−^ emission, and reducing the amount of all types of PL quenchers in the system, and hence, substantially improving the crystal quality of particles with sizes exceeding a dozen nanometres [34, 35]. Further works on these topics are now in progress.

### DND Aggregate-Specific Fluorescence and Discrimination of Diffraction-Limited Emitters

Characterizing the fluorescence from isolated DND particles or submicron aggregates is crucial both for understanding their potential use as fluorescent markers and to help to mitigate disadvantages related with a relatively low concentration of NV^−^ in them as determined by EPR. Photoluminescence was recorded for DND aggregates spin-coated on a glass microscope coverslip from an aqueous suspension with an average size of DND aggregates about 30 nm (as measured by dynamic light scattering).

**Fig. 7:**
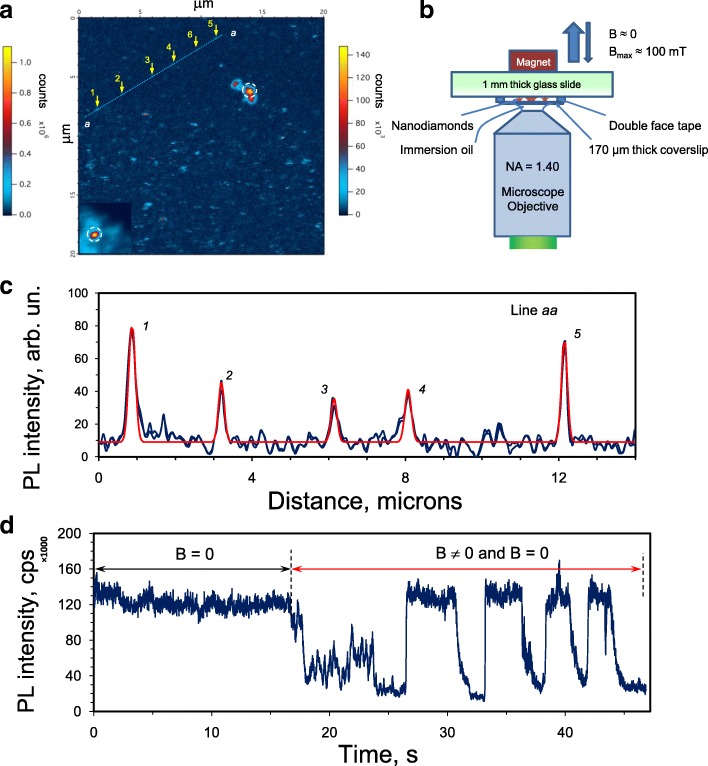
**a** 2D-colour mapping of the PL signal intensity of DND spin-coated on a glass microscope coverslip together with **b** schematics of the experimental setup. **c** The PL intensity profile along the “*aa”* cross-section. **d** The photoluminescence intensity versus time for the selected DND aggregate marked with a circle in the upper side of the 2D map shown in panel **a**, in the presence or absence of an external magnetic field. Laser excitation at 532 nm wavelength. Square, 201 × 201 pixels. Integration time is 3 ms/pixel. Step—100 nm. The excitation radiation is focused on the upper surface of the glass coverslip with deposited DND. In zero field (*B* = 0), only small changes of intensity, due to the blinking of some of the NV^−^ colour centres occur. When magnetic field is temporally applied a large decrease of the PL intensity occurs. Upper left panel **a**: the left scale for PL intensity corresponds to the 2D mapping of another DND aggregate shown in the left bottom corner of the large 2D map. Upper right panel **b**: schematics of the experimental setup explaining the displacement of the permanent magnet above the coverslip relative to the deposited DND

Figure 7a shows two PL 2D maps obtained by confocal microscopy, using a 532-nm wavelength excitation laser with 100-μW output optical power. Bright spots corresponding to DND aggregates up to 500–700 nm in lateral size are observed. Dimmer spots of size around the optical diffraction limit are also observed after selection of the appropriate isolated spots. Figure 7c shows the intensity distribution along the specially selected straight line *aa* plotted in Fig. 7a. This line crosses about six dim spots of smallest diameter—five spots (1–5) are crossed by the straight line fairly precisely along their centres and one spot (6) is with a small displacement from this line. The corresponding five peaks in intensity distribution are clearly seen in Fig. 7c. Thus, each dim spot 1–5 laying on the line *aa* corresponds to the DND aggregate of smallest lateral size (in the range below 70 nm). It is possible that all of them are fixed on one V-shaped straight groove existing on the glass coverslip. We successfully fitted each peak in the intensity distribution with a 2D Gaussian ${A}_i{e}^{-\left[{\left(x-{x}_{oi}\right)}^2+{\left(y-{y}_{oi}\right)}^2\right]/2{s}_i^2}$, where *x*_*oi*_, *y*_*oi*_ are the Cartesian coordinates of the centres of the dim round spots, *A*_*i*_-maximum PL intensity of each isolated spot, *s*_*i*_ is a parameter close to *s*_*o*_ ≈ *r*_o_/3, where *s*_*o*_ is a 1/3 part of the Airy disk diameter *r*_o_. In our case, for *λ* = 532 nm radiation (in vacuum) and numerical aperture of microscopic objective NA = 1.40, we have the following values for *r*_o_ and *s*_o_: *r*_o_=1.22λ/2NA ≈ 232 nm and *s*_o_ *≈* 77 nm*.* The *s*_o_ value in the 2D Gaussian determines the point spread function (PSF) of and ideal point-like emitter, i.e. the diffraction limitation related with the smallest possible interference ring. For the five peaks mentioned above (*i* = 1–5) we found the following *s*_*i*_ values, respectively: 85, 77, 77, 84 and 77 nm. Peak 5 has both the highest intensity and *s*-value equal to the theoretical value *s*_o_ *≈* 77 nm. This means that the lateral size of the corresponding emitter for peak 5 is significantly smaller than the PSF size. The same is also true for peaks 1–4. We can conclude that the lateral sizes of DND aggregates laying along line *aa* are in the range below 70 nm. Each spherically shaped DND aggregate of ~ 30 nm in size^5^ consisting of individual 5-nm particles (percentage of voids is 50%) has about 1.3 NV^−^centres in accordance with the 1.1 ppm content of NV^−^ determined previously. This value is great enough in principle to distinguish aggregates of this size by optical methods. Each of the five aggregates lying along the line *aa* probably has between 2 and 10 colour centres. The larger the height of the DND aggregate lying on the substrate, the higher the density of luminescence centres (per unit square) for the light emitted more or less perpendicular to the surface. For aggregates with height up to 300–350 nm, the brightness of PL intensity can be at least one order of magnitude greater than that for aggregates of smaller 25–30 nm size. The PL spectrum of one selected aggregate of high brightness (marked by a circle in the 2D map presented in Fig. 7a) was studied in detail. It has roughly the same shape (especially on the right wing above 680 nm) as that for the spectrum shown in Fig. 6a and indicates the presence of both NV^−^ and NV^0^ defects. However, the characteristic zero-phonon lines (ZPL) at 638 nm (NV^−^) and 575 nm (NV^0^), respectively, were again not detected. ZPLs are usually well resolved only for diamond crystals of micron size or larger, while for nanometre-scale crystals, they are typically not well-observed due to some experimental or other physical reasons^6^. Let us mark that for DND aggregates lying on the cover glass, the final treatment with UV/O-Cleaner removed the *sp*^2^-coordinated carbon species around the particles inside the aggregates and as a result the PL spectrum is free from the contribution of light-emitting centres of disorganized *sp*^2^-carbon phase.

To confirm the presence of NV^−^ centres in the DND, we studied the influence of an external magnetic field on the PL intensity. No PL modification is expected from NV^0^, which have no magneto-optical properties unlike NV^−^. Figure 7d shows the meander-like time variation of the PL intensity from an isolated DND aggregate in the presence of an external magnetic field switched “ON” by quickly bringing a compact permanent magnet close to the DND or by removing it (“OFF”). This occurs as a result of the mixing of the |0〉_GS_ and |−1〉_GS_ states of NV^−^ centres at the ground-state spin level anti-crossing (GSLAC), marked with a circle at magnetic field ~ 100 mT in Fig. 3. Such mixing leads to a change in the populations of these states, accompanied by a PL intensity decrease. The optical transitions between the ground ^3^A_2_ and excited ^2^E triplet states preserve the spin quantum number (Δ*m*_*S*_ = 0). However, from the *m*_*S*_ = ± 1 excited state, the optical excitation of NV^–^ also decays with no radiation in the visible domain, through a system of two metastable singlet states before coming back to the ground state [5]. This process is accompanied by radiation at a longer wavelength of 1042 nm defined by the gap between these two singlet levels with *S* = 0. This additional decay pathway results in a lower fluorescence intensity from the main radiation transition within the *m*_*S*_ = ± 1 subsystem of ground and excited triplet states. The experimentally detected decrease of NV^–^ centre PL intensity is quite reasonable in the presence of a weak (≤ 100 mT) magnetic field [36], as observed in Fig. 7d. Surprisingly, in our case (for DNDs), this drop is essentially larger than those reported in the literature for Ib HPHT fluorescent microdiamonds and even evaluated theoretically in the framework of the standard NV model [37].

## Conclusion

In this article, we have shown that nitrogen-vacancy centres appeared in the interior of 5 nm nanodiamond particles synthesized by detonation of nitrogen-containing explosives are the main triplet colour centres. Precise counting of the number of NV^−^ centres was achieved. The DNDs contain ~ 1.1 ppm of such NV^−^ centres in the crystalline diamond lattice. This value is just five times smaller than that detected in the bright fluorescent reference Ib HPHT diamonds with a mean size of about ~ 100 nm. Our adjusted and rechecked estimation made on the basis of the EPR method is about three orders of magnitude larger than that evaluated in Ref. [38] by pure optical methods for DND aggregates. Probably not all NV^−^ centres detected by EPR are optically active due to the NV^−^ luminescence quenching by various point and collective defects located near the surface. Annealing DNDs in oxygen- or ozone-containing air removes defects associated with light-absorbing *sp*^2^ carbon at the particle surface and allows detection of the specific PL of NV^−^ centres. When an external magnetic field is applied, an accompanying variation of the PL intensity is observed. This is the result of the sensitivity of the NV^−^ triplet ground state to magnetic field and comes through the mixing of the |0〉_GS_ and |−1〉_GS_ states of only the NV^−^ centres below/near the GSLAC point leading to the decay of the optical excitation with no radiation in the visible domain. The application of this effect to discriminate ultra-small DND emitters in an environment with a large autofluorescence background and micron-scale bio-object contouring is promising [39]. Some DND aggregates playing the role of point-like NV^−^ emitters and giving the smallest fluorescent spots with diameter close to the PSF size (diffraction limitation) were found.

## Abbreviations

2D: Two dimensional
CCD: Charge-coupled device
DND: Detonation nanodiamond
EPR: Electron paramagnetic resonance
FND: Fluorescent nanodiamond
GSLAC: Ground state spin levels anti-crossing
HPHT: High pressure high temperature
NA: Numerical aperture
NV: Nitrogen-vacancy
ODMR: Optically detectable magnetic resonance
PL: Photoluminescence
PSF: Point spread function
SCTB: Special design and technology bureau
SQUID: Superconducting quantum interference device
UV: Ultraviolet
XPS: X-ray photoelectron spectroscopy
XRD: X-ray diffraction
ZPL: Zero-phonon line

## Footnotes

1 The same data for concentration of paramagnetic centres *S* = ½ (≥ 1260 ppm) were obtained independently by precision measurements of the magnetization field dependence at temperature *T* = 2 K using a superconducting SQUID magnetometer. The magnetization curve does not show the presence of spins 3/2 in appreciable quantity in DNDs [13].

2 See for example the details of this method for analysis of the standard (DND) and non-standard (NDB-G) samples given in Ref. [22].

3 Fluorescent Ib HPHT (high pressure high temperature) microcrystals doped with nitrogen (100–150 ppm) and thereafter irradiated by high energy electrons (2–15 MeV) and annealed at 800–950 ^o^C.

4 We assume that a typical DND particle (with size slightly exceeding 5 nm) consists of ~ 1.1 × 10^4^ carbon atoms.

5 Here we consider the typical DND aggregate in water DND suspension used for deposition the aggregates on the glass coverslip. Certainly, the actual shape of aggregates lying on the cover slip can be rougher and extremely diverse depending upon the adhesion of DND particles to the glass and actual size of the large-scale aggregates.

6 The ZPL line at 638 nm was not resolved in many works where the nanodiamonds having NV^−^ were studied [4, 31]. It is probable that the various local environments of NV^−^ centres in nanosized diamond (like surface defects and interior imperfections) greatly disturb the position of NV^−^ triplet ground states in the bandgap.
